# Genetic dissection of powdery mildew resistance in interspecific half-sib grapevine families using SNP-based maps

**DOI:** 10.1007/s11032-016-0586-4

**Published:** 2016-12-21

**Authors:** Soon Li Teh, Jonathan Fresnedo-Ramírez, Matthew D. Clark, David M. Gadoury, Qi Sun, Lance Cadle-Davidson, James J. Luby

**Affiliations:** 1 Department of Horticultural Science, University of Minnesota, Saint Paul, MN 55108 USA; 2 Department of Horticulture and Crop Science, The Ohio State University/OARDC, Wooster, OH 44691 USA; 3 School of Integrative Plant Science, Cornell University, New York State Agricultural Experiment Station, Geneva, NY 14456 USA; 4 BRC Bioinformatics Facility, Institute of Biotechnology, Cornell University, Ithaca, NY 14853 USA; 5 USDA-ARS Grape Genetics Research Unit, Geneva, NY 14456 USA

**Keywords:** Half-sib progeny, Interspecific, Grapevine, Powdery mildew, Pseudo-testcross, QTL

## Abstract

Quantitative trait locus (QTL) identification in perennial fruit crops is impeded largely by their lengthy generation time, resulting in costly and labor-intensive maintenance of breeding programs. In a grapevine (genus *Vitis*) breeding program, although experimental families are typically unreplicated, the genetic backgrounds may contain similar progenitors previously selected due to their contribution of favorable alleles. In this study, we investigated the utility of joint QTL identification provided by analyzing half-sib families. The genetic control of powdery mildew was studied using two half-sib F_1_ families, namely GE0711/1009 (MN1264 × MN1214; *N* = 147) and GE1025 (MN1264 × MN1246; *N* = 125) with multiple species in their ancestry. Maternal genetic maps consisting of 1077 and 1641 single nucleotide polymorphism (SNP) markers, respectively, were constructed using a pseudo-testcross strategy. Ratings of field resistance to powdery mildew were obtained based on whole-plant evaluation of disease severity. This 2-year analysis uncovered two QTLs that were validated on a consensus map in these half-sib families with improved precision relative to the parental maps. Examination of haplotype combinations based on the two QTL regions identified strong association of haplotypes inherited from ‘Seyval blanc’, through MN1264, with powdery mildew resistance. This investigation also encompassed the use of microsatellite markers to establish a correlation between 206-bp (UDV-015b) and 357-bp (VViv67) fragment sizes with resistance-carrying haplotypes. Our work is one of the first reports in grapevine demonstrating the use of SNP-based maps and haplotypes for QTL identification and tagging of powdery mildew resistance in half-sib families.

**Electronic supplementary material:**

The online version of this article (doi:10.1007/s11032-016-0586-4) contains supplementary material, which is available to authorized users.

## Key message

Analysis of pedigree-connected, multi-parental grapevine families having multi-species ancestry enabled QTL detection and validation, and construction of SNP-based haplotypes associated with powdery mildew resistance in a cold-climate breeding program.

## Introduction

Powdery mildew, caused by the biotrophic ascomycete *Erysiphe necator*, is a serious fungal disease of grapevine (*Vitis* spp.) worldwide (Gadoury et al. 2012). The fungus was inadvertently introduced to Europe through importation of grape cuttings from North America during the mid-nineteenth century. Subsequently, it was rapidly spread to other regions where grapevines were cultivated. Cultivated Eurasian grapevine (*Vitis vinifera* L.) is susceptible to the fungus, which can infect all green tissues as well as inflorescences and immature fruit, resulting in yield losses due to reduced photosynthetic leaf area, poor fruit set, premature fruit drop, and diseased berries (Pearson 1988).

Control of powdery mildew typically relies on frequent application of fungicides, which is becoming increasingly prohibitive due to their cost and adverse effects on human health and the environment. With modern grapevine cultivation moving toward sustainable practices, grape breeding for disease resistance offers potentially effective and environment-friendly control of powdery mildew. Marker-assisted breeding can expedite cultivar improvement for disease resistance by predicting the presence of resistance alleles when selecting parents or offspring.

Natural sources of disease resistance are typically found in regions where the pathogen and the host plant populations co-evolved. In particular, grapevine germplasm native to the North American and East Asian regions have been evaluated by breeders for sources of powdery mildew resistance. Thus far, the genetic research on powdery mildew resistance has elucidated quantitative trait locus (QTL) from several genetic backgrounds, including *V. vinifera*, Asian *Vitis* species, and hybrids of North American *Vitis* species, as well as *Muscadinia rotundifolia* (Michx.) Small, also known as *Vitis rotundifolia* Michx. (Barker et al. 2005; Blanc et al. 2012; Dalbó et al. 2001; Hoffmann et al. 2008; Pap et al. 2016; Ramming et al. 2011; Riaz et al. 2011; Welter et al. 2007).

Genetic mapping in highly heterozygous, outbreeding crops like grapevine typically requires the use of a mapping strategy termed “pseudo-testcross” for construction of linkage maps (Grattapaglia and Sederoff 1994). In a conventional testcross where a heterozygous individual is crossed with an inbred that is homozygous at all loci, subsequent segregation can be attributed to the heterozygous parent. Meanwhile, a pseudo-testcross strategy implements crossing of two highly heterozygous individuals to identify markers that are fully informative with respect to the segregation attributed to the parent of interest. Despite being restricted by the number of usable markers with the approach, the inclusion of multiple mapping families provides an opportunity to harness markers heterozygous for both parents of a full-sib family for the eventual construction of a multiple-family consensus linkage map.

The advancement of whole-genome sequencing and next-generation sequencing has propelled crop genomic and genetic research. In particular, genotyping-by-sequencing (GBS) offers an inexpensive and robust solution for simultaneous single nucleotide polymorphism (SNP) discovery and genotyping through pooled, barcoded, reduced representation libraries, sequencing, and SNP assignment based on alignment of short reads (Elshire et al. 2011). The results are thousands of low-coverage markers that are usable for QTL mapping in bi-parental families (Davey et al. 2011). The work by Barba et al. (2014) demonstrated the first application of the GBS procedure in generating SNP markers to construct a high-resolution map for QTL mapping in grapevine.

Breeding programs in cold-climate regions typically utilize recurrent selection to improve traits, such as maturity, winter hardiness, fruit quality, and disease resistance. In perennial fruit crops such as grapevine, funding, labor, and space constraints due to plants’ large size and long generation time may hamper such efforts. In this breeding method, clonal propagation and the long life of grapevines allows breeders to use desirable breeding parents to contribute their favorable alleles in multiple diverse crosses. Hence, the availability of multiple experimental families having pedigree-connected parents provides an opportunity to explore the genetic control of traits using a pseudo-testcross approach that combines information from parental meioses over multiple families. Genetic mapping studies using related existing families could be a useful tool in grapevine for (1) identifying reliable QTL and (2) developing markers that are relevant to the breeding germplasm.

Hence, the main objectives of this investigation were to (1) build SNP-based linkage maps for interspecific experimental *Vitis* families using pseudo-testcross strategy, (2) construct a consensus linkage map using markers commonly present in the maternal (MN1264) maps of these half-sib families, (3) identify and validate QTL for powdery mildew resistance in both parental and consensus maps, (4) develop SNP-based haplotypes that are associated with resistance, and (5) identify linked simple sequence repeat (SSR) markers that may be useful for future crossing or culling decisions in a breeding program.

## Materials and methods

### Plant material

**Fig. 1:**
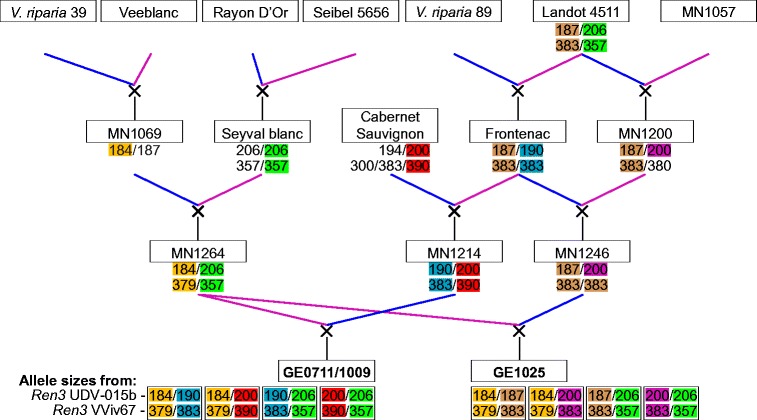
Two-generation pedigrees and ancestral species backgrounds of two mapping families, GE0711/1009 and GE1025. Pedigree information was obtained from the breeding records of *Vitis* International Variety Catalog, VIVC (https://www.vivc.de/). Allele sizes from *Ren3* SSR markers were colorized to trace the inheritance of alleles

Two bi-parental F_1_ families were used in this study (Fig. 1). A cross of MN1264 × MN1214 made in 2007 (GE0711; *N* = 53) and was repeated in 2010 (GE1009; *N* = 94). These crosses were treated as a single mapping family, GE0711/1009, for this experiment. The second mapping family, GE1025 was a cross of MN1264 × MN1246 made in 2010 (*N* = 125). Comprised of at least six *Vitis* species in their ancestry, including *V. vinifera*, *Vitis riparia*, *Vitis rupestris*, *Vitis labrusca*, *Vitis aestivalis*, and *Vitis berlandieri*, these F_1_ families were grown at the University of Minnesota Horticultural Research Center (44° 52′ 08.1″ N, 93° 38′ 17.3″ W). They were chosen as mapping populations for the USDA-funded *Vitis*Gen project because their pedigrees are connected and contain many key ancestors contributing winter hardiness and fruit quality to the University of Minnesota wine grape breeding program.

### Quantification of field powdery mildew severity

Throughout the 2-year investigation, no fungicide applications were used. Disease severity on each vine was evaluated toward the end of each growing season. In late August of 2014, each vine was assessed using a 7-point visual scale of whole-plant foliage (1 = absent, 3 = sporulation covers up to a third of the foliage, 5 = sporulation covers up to two thirds of the canopy, 7 = vast and dense sporulation covers over two thirds of the canopy; even numbers denote intermediate symptoms). In 2015, the leaves of each vine were evaluated using the 9-point International Plant Genetic Resources Institute (IPGRI) scale that was established by the Organization Internationale de la Vigne et du Vin (IPGRI et al. 1997) on August 27 (1 = absent, 3 = low sporulation with limited patches <2 cM in diameter, 5 = moderate sporulation with patches of 2–5 cM in diameter, 7 = vast sporulation and abundant mycelium, 9 = extreme sporulation with unlimited patches of sporulation; even numbers denote intermediate symptoms).

In GE1025, field phenotype data were collected throughout the growing season after the first observation of powdery mildew. Plants were evaluated every 7 to 10 days until the disease severity of the mapping family reached a plateau. Observation of disease symptoms over the multiple time points allowed the construction of a disease progress curve (DPC) for QTL mapping to determine if area under disease progress curve (AUDPC) provided additional utility and resolution in QTL detection. In 2014, GE1025 plants were visually evaluated on July 24, July 31, August 14, August 26, September 5, September 12, September 19, and September 26. In 2015, field ratings were collected on June 19, June 26, July 2, July 10, July 17, July 23, July 30, August 6, August 13, and August 20 (Fig. S2).

### DNA extraction, sequencing, and SNP assignment

Methods from tissue sampling through SNP assignment were as described previously (Hyma et al. 2015). For each vine, a single, small, newly expanding leaf (less than 1-cm diameter) was collected in one tube of a Costar 96-well cluster tube collection plate (Corning Life Sciences, Tewksbury, MA, USA). Leaf tissues were maintained at 4 °C from harvest until processing in the laboratory where two stainless steel genogrinder beads were placed in each tube and plates were frozen at −80 °C. Tissue grinding took place in a Geno/Grinder 2000 (OPS Diagnostics LLC, Lebanon, NJ, USA) with 96-well plates agitated in pairs at 400× speed for 1 min. Plates were then stored at −80 °C until processing with DNeasy 96-well DNA extraction kits (QIAGEN, Valencia, CA, USA). Modifications were made to the manufacturer’s protocol to improve DNA quality and quantity as follows: (1) PVP-40 (2 % *w*/*v*) was added to the AP1 lysis buffer prior to heating and (2) visual inspection for complete re-suspension of the sample pellet of each 8-tube strip was added to the agitation step following AP1 addition.

GBS was performed as described by Elshire et al. (2011), integrating four 96-well plates across 384 bar codes for library preparation (Hyma et al. 2015) and sequencing of single-end 100-bp sequences using a HiSeq 2000 (Illumina Inc., San Diego, CA, USA). Raw data for the University of Minnesota germplasm (henceforth, UMN dataset) was processed based on an updated version of the *Vitis*Gen databased previously employed by Hyma et al. (2015). Comprised of 409 maternal half-sib individuals and four progenitors, the UMN dataset was processed through TASSEL 3.0.139 GBS pipeline (Glaubitz et al. 2014) using the 12×.2 *V. vinifera* “PN40024” reference genome (Adam-Blondon et al. 2011; Jaillon et al. 2007) from The French-Italian Public Consortium (https://urgi.versailles.inra.fr/Species/Vitis/Data-Sequences/Genome-sequences). For alignment, Burrows–Wheeler Aligner maximal exact match (BWA-MEM) was applied using with default parameters (Li and Durbin 2009). The output consisted of variant call format (VCF) file version 4.1 (Danecek et al. 2011) including SNPs present in at least 40 % of the progeny, and with a minor allele frequency (MAF) ≥0.1. Subsequently, the VCF file was filtered using vcftools ver. 1.12a (Danecek et al. 2011) and TASSEL (Bradbury et al. 2007) versions 3.0.139 and 4.3.13. Finally, the VCF file was sliced to include SNP data of the 277 F_1_ individuals (in mapping families of GE0711/1009 and GE1025) and the three progenitors (MN1264, MN1214, and MN1246) in this study.

### Linkage maps

From the putative markers identified for GE0711/1009, GE1025, and the progenitors, a custom filtering process for alignment was applied, with minimum read depth of 6 and 85 % completeness by site across progeny and by progeny across sites. Subsequently, an additional filter for MAF between 0.05 and 0.35 was applied through vcftools to optimize the identification of markers for parental map construction. Results were output as a TASSEL hapmap file. Pseudo-testcross markers with segregation of 1:1 (heterozygous/homozygous) were identified by a chi-squared (χ^2^) goodness-of-fit test at *α* < 0.01, coded and imported into JoinMap 4.1 (Van Ooijen 2006).

Using a two-way pseudo-testcross approach (Grattapaglia and Sederoff, 1994) and cross-pollination (CP) population in JoinMap, marker types of *lm × ll* and *nn × np* were retained for construction of maternal and paternal maps, respectively. Each parental linkage map was constructed separately. As an additional level of stringency, markers with over 10 % missing data were discarded. Linkage groups (LGs) were constructed with a minimum threshold logarithm of odds (LOD) score of 6.0. LGs were grouped and numbered based on their corresponding physical chromosome numbers (Jaillon et al. 2007). Genetic maps and marker order were determined using the regression mapping algorithm with Haldane function and default parameters. Initial maps obtained in JoinMap were further analyzed in R/qtl software (Broman et al. 2003) using a four-way cross format. Markers were assessed for segregation distortion using geno.table command at a *p* value of 1 × 10^−7^, above which markers were discarded. Finally, map distance and marker order were recalculated in JoinMap.

### Construction of a consensus genetic map

Development of the maternal (MN1264) consensus map was conducted using LPmerge (Endelman and Plomion 2014). Contrary to the approach of minimizing an objective function based on the observed recombination frequencies between markers, this software employs a linear programming algorithm to minimize the mean absolute error between the consensus map and the genetic maps from each family. This minimization step results in preservation of marker order in linkage maps. At instances where marker order is inconsistent between maps, LPmerge implements an additional algorithm to resolve ordinal conflicts.

The LPmerge software contains two key parameters that are set by the user: (1) maximum interval size between bins and (2) weights applicable to each map. In our analysis, the maximum interval parameter was set at *K* = 8, while weights were adjusted based on family sizes due to more progeny providing better resolution. At each LG, the best consensus map was selected using two criteria: (1) minimization of the average root-mean-squared error (RMSE), and (2) map length comparable to the mean of the linkage maps. Final images of LGs were generated using MapChart 2.2 (Voorrips 2002) before they were exported to Adobe Illustrator CS2 for alignment and vector transformation.

### QTL analysis

QTL detection was conducted using multiple statistical analyses and software: Kruskal-Wallis (KW), interval mapping (IM), and composite interval mapping (CIM). KW and IM were performed on MapQTL 6 software (Van Ooijen and Kyazma, 2009), which implemented a maximum likelihood mixture model. Meanwhile, CIM was analyzed in R/qtl software (Broman et al. 2003) using default parameters. The minimum LOD score required for QTL detection was approximately 3, which was determined by the genome-wide LOD significance threshold (*α* = 0.05) calculated using 1000 permutations.

### Haplotype construction and analysis with *Ren3* SSRs

Based on the maternal consensus map, 12 and 22 SNP markers spanning the QTL (described in “Results”) on LG 15 and LG 2, respectively, were used to build the grapevine powdery mildew resistance haplotypes. With the use of SNPs from a pseudo-testcross approach, there are two unique haplotypes for each QTL spanned, thereby yielding a total of four haplotype combinations for the two QTLs of interest. Given the availability of GBS information for the maternal grandparents (i.e., MN1069 and ‘Seyval blanc’) of the progeny, the haplotypes were traced based on inheritance and were correspondingly assigned.

Analysis of variance (ANOVA) calculations were made to determine if a haplotype combination was associated with disease severity in each year. Where statistical difference was detected, Tukey’s honest significant difference (HSD) test (*p* < 0.05) was conducted as a post hoc analysis to test differences among the average effects of each haplotype combination.

The identification of powdery mildew resistance QTL on LG 15 raised the possibility of co-localization of this QTL with the previously reported *Ren3* (Welter et al. 2007). Two SSR markers (UDV-015b and VViv67) for powdery mildew resistance that were associated with the *Ren3* resistance locus were used to screen the mapping families and the progenitors. According to Welter et al. (2007), these markers were reported to be 3 cM apart and are located in the vicinity of *Ren3*. The amplified fragment sizes were used to trace the inheritance of allele sizes in the known grandparent-parent-offspring relationships (Fig. 1).

### Haploblock validation using a controlled inoculation experiment

In addition to obtaining field phenotype data based upon natural powdery mildew infection, controlled inoculation of GE1025 mapping family was carried out using *E. necator* isolate NY19 in two independent experiments (experiment A: *N* = 119; experiment B: *N* = 106). Protocols for the maintenance of pathogen isolate, leaf sampling, inoculation, and microscopic evaluation at 9-day post infection (9 dpi) were described in Cadle-Davidson et al. (2016). At 9 dpi, leaf samples were evaluated based on several responses: (i) number of hyphae intercepting a transect through a ​200× field of view, henceforth referred to as hyphal transects; (ii) cube root transformation of hyphal transects, henceforth annotated as TransectT; and (iii) incidence of powdery mildew sporulation. Using the different haplotype combinations that were constructed based on the QTL regions, haploblock validation for each phenotypic response was carried out to determine the effect of each haplotype. Statistical analyses were conducted in JMP 12.0.1 (SAS Institute Inc. Cary, NC, USA).

## Results

### Genotyping: sequencing and SNP assignment

The SNP assignment conducted in TASSEL 3.0.139 produced a total of 526,775 SNP markers in the UMN dataset. The markers were the result of successful alignment of 595,270 GBS tags from a total of 886,142 (67.18 %), with no alignment to multiple positions being reported in this dataset. With respect to the mapping families and progenitors in this study, the total number of SNPs from the UMN dataset was retrieved for subsequent filtering. Preliminary filtering (≥40 % marker presence in the progeny; MAF ≥0.1) yielded 103,072 markers for GE0711/1009 and 82,324 markers for GE1025. For parental maps, 5464 SNP markers suitable for pseudo-testcross analysis were identified for GE0711/1009, and 5738 SNPs for GE1025.

### Parental and consensus linkage maps

Maternal (MN1264) genetic maps of GE0711/1009 and GE1025 were constructed using 1077 and 1641 SNPs each, covering genetic distances of 2142 and 2005 cM, respectively, and physical distances of approximately 426.2 Mb (Fig. S4. All significant markers (LOD score ≥6.0) were included in JoinMap 4.1 for linkage map construction, omitting unlinked markers due to weak linkages to other markers within the LG (Tables 1, S1). Information of the paternal maps (MN1214 and MN1246) is summarized in Table S1, as well as in Figs. S5 and S6. The use of the LPmerge software to build a consensus map provided LGs with comparable genetic lengths. The consensus map was constructed using 1977 SNPs, covering a total genetic distance of 1852.9 cM with an average interval of 0.94 cM between markers (Table 1).

**Table 1:** Number of SNPs and total genetic distance (cM) of linkage groups (LGs) in the common parent MN1264 genetic maps of mapping families, GE0711/1009 (*N* = 147) and GE1025 (*N* = 125)

LG	GE0711/1009		GE1025		Consensus	
	Number of SNPs	Total genetic distance (cM)	Number of SNPs	Total genetic distance (cM)	Number of SNPs	Total genetic distance (cM)
1	26	32.5	53	29.2	54	34.0
2	59	97.4	67	74.5	93	90.2
3	31	95.0	47	91.6	51	56.3
4	87	145.1	99	87.5	148	130.1
5	75	109.7	141	120.4	153	109.7
6	51	78.6	76	98.6	90	97.0
7	87	132.4	133	137.7	165	137.7
8	78	104.7	89	80.3	126	84.7
9	48	112.1	60	82.1	73	82.1
10	40	71.9	57	78.1	65	77.7
11	30	97.9	47	92.6	52	93.8
12	63	108.9	78	91.9	112	104.4
13	80	125.5	118	144.4	149	70.9
14	54	140.9	135	115.3	151	139.4
15	64	129.8	111	111.9	123	122.7
16	36	100.6	49	144.7	61	97.6
17	37	113.2	91	94.4	94	106.6
18	82	238.9	113	189.6	127	106.6
19	49	107.3	77	140.6	90	111.4
Total	1077	2142.4	1641	2005.4	1977	1852.9

Generally, the marker order between the consensus map and the parental maps was conserved. In most intervals, markers in the consensus map were co-linear to those in the two parental maps. Peculiarly, a region of 13 markers spanning from 7.2 to 39.9 cM on LG 15 of the maternal map of GE0711/1009 was mapped onto just a single locus at 24.4 cM on the consensus map. Since these markers were not in common with any SNP on GE1025, this could be a rare instance where LPmerge was unable to resolve the recombination events that originated from GE0711/1009.

### Field assessment of powdery mildew severity

Disease phenotypes of progeny in the two experimental families spanned nearly the complete rating scale in both years (Fig. S1), with positive correlation between the 2 years (Pearson’s *R* ^2^ = 0.60; Spearman’s *R* ^2^ = 0.60). In 2014, the mean scores were 4.1 in GE0711/1009 (*N* = 147) and 4.7 in GE1025 (*N* = 125) based on a 7-point visual scale. Meanwhile, in 2015, the mean scores for the families were 4.7 and 5.3, respectively, based on the 9-point IPGRI scale.

In the construction of a DPC, field evaluation of GE1025 started over a month earlier in 2015 than in 2014. In general, the vast majority of individuals were rated between one and three during the first 21 days of disease assessment. Field ratings of GE1025 appeared to level off approximately 55 days after the initial assessment.

### Analysis of marker-trait association

QTL mapping was conducted using IM and CIM on each maternal map, and resulted in identification of QTL on LGs 2 and 15 (Table 2; Fig. S3). In GE0711/1009, a moderate QTL on LG 2, and a major QTL on LG 15 were detected using 2014 ratings and supported by significant LOD scores at chromosome- and genome-wide thresholds (*α* = 0.05). Though a QTL on LG 2 was again detected in GE0711/1009 using 2015 ratings, no significant QTL was detected on LG 15. In GE1025, a QTL was identified in the same genetic region of LG 15 in both years and explained a similar proportion of phenotypic variance, regardless of the analysis methods. In contrast to GE0711/1009, a QTL was not detected on LG 2 in either year. In the QTL analysis using the consensus map, a minor QTL on LG 2 was detected in 2015, but not in 2014. Meanwhile, results of QTL analysis for LG 15 were consistent across years, where a major QTL was mapped to the region between 12.0 and 24.0 cM (CIM) with peak LOD score of approximately 10. As a whole, the use of the more sensitive CIM analysis method identified QTL that generally peaked at a similar genetic position in both years, and reported a finer two-LOD confidence interval overlapping the interval from IM.

**Table 2:** Summary of 2-year QTL information for powdery mildew resistance identified on LGs 2 and 15 for linkage maps of GE0711/1009 (*N* = 147) and GE1025 (*N* = 125), as well as the consensus map (*N* = 272)

LG	Map	Year	Analysis	LOD threshold		LOD_max_	LOD_max_ position (cM)	Variance explained (%)	Confidence interval (cM) [LOD_max_ − 2]
				LG-specific^a^	Genome-wide^a^				
2	GE0711/1009	2014	IM	1.60	3.10	3.46	82.2	11.7	67.8–97.4
			CIM	–	3.68	3.49	83.0	10.1	77.0–86.1
		2015	IM	1.50	2.90	4.21	79.0	13.3	71.2–94.5
			CIM	–	3.73	5.38	79.0	13.3	74.8–84.0
	GE1025	2014	IM	4.10	4.10	–	–	–	–
			CIM	–	4.00	–	–	–	–
		2015	IM	6.50	6.50	–	–	–	–
			CIM	–	4.75	–	–	–	–
	Consensus	2014	IM	1.80	3.20	–	–	–	–
			CIM	–	3.54	–	–	–	–
		2015	IM	1.90	3.30	4.19	79.6	7.2	67.1–90.2
			CIM	–	3.58	3.91	74.8	7.0	69.0–78.0
15	GE0711/1009	2014	IM	1.60	3.10	5.58	7.2	17.3	0.0–58.5
			CIM	–	3.68	6.79	10.0	16.1	2.0–11.0
		2015	IM	1.60	2.90	–	–	–	–
			CIM	–	3.73	–	–	–	–
	GE1025	2014	IM	1.80	4.10	6.32	16.9	21.4	2.0–27.9
			CIM	–	4.00	8.08	16.9	20.1	12.0–21.2
		2015	IM	1.70	6.50	9.08	5.5	29.4	1.0–29.5
			CIM	–	4.75	10.30	14.0	28.5	9.0–18.0
	Consensus	2014	IM	1.60	3.20	10.21	18.5	17.1	5.0–24.4
			CIM	–	3.54	10.57	19.4	16.0	12.0–21.2
		2015	IM	1.70	3.30	9.11	16.5	15.7	2.0–37.2
			CIM	–	3.58	9.83	18.0	15.0	15.0–24.0
	GE1025 (AUDPC)	2014	IM	1.70	3.90	7.59	5.54	25.6	2.0–46.4
			CIM	–	4.14	6.96	13.2	23.6	5.5–15.0
		2015	IM	1.80	3.50	8.23	5.54	27.0	0.0–51.4
			CIM	–	4.04	8.21	5.54	27.0	1.0–10.5

^a^Estimated threshold values using a permutation test with 1000 permutations at *α* = 0.05

The use of AUDPC as a phenotype for QTL mapping corroborated the QTL findings based on a single time-point phenotype (Table 2). In both years, AUDPC in GE1025 allowed the detection of a major QTL on LG 15, explaining up to 27.0 % of phenotypic variance. Similar to the QTL results from a single time-point phenotype, AUDPC in GE1025 also did not detect a QTL on LG 2.

### Haplotypes associated with powdery mildew resistance

Haplotypes were constructed using 12 SNPs (covering ∼6.6 cM) and 22 SNPs (covering ∼24.3 cM) that spanned the LG 2 and LG 15 QTL regions, respectively. Linkage phases of the haplotypes for the maternal parent (MN1264) were determined; there were no observable recombination events from the grandparent progenitors, ‘Seyval blanc’ and MN1069. Considering only the maternal grandparent origin for LGs 2 and 15, four haplotype combinations are possible, namely LG02_MN1069_/LG15_MN1069_, LG02_Seyval blanc_/LG15_MN1069_, LG02_MN1069_/LG15_Seyval blanc_, and LG02_Seyval blanc_/LG15_Seyval blanc_.

**Fig. 2:**
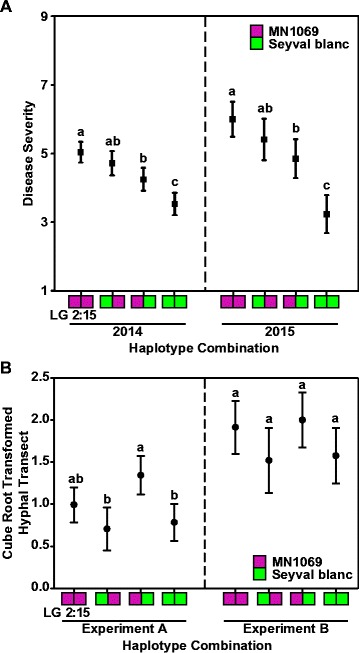
**a** Correlation between disease severity and four haplotype combinations in the combined maternal families of GE0711/1009 and GE1025 showed evidence of genotypes with ‘Seyval blanc’-inherited haplotypes having the lowest disease severity mean score in both years. **b** Correlation between colonization (expressed in cube root transformed hyphal transect) and four haplotype combinations in GE1025 showed evidence of individuals with ‘Seyval blanc’-inherited *Ren10* haplotype exhibiting the lowest mean of colonization in experiment A. Similar trend was shown in experiment B, but lacked statistical support. Different *letters* were assigned on score ranges to indicate statistical significance that was computed by Tukey’s HSD test (*p* < 0.05)

Analyses of the four haplotype combinations revealed three statistically different classes for disease severity (Fig. 2a; Table S2). Progeny with MN1069-inherited haplotypes consistently exhibited the highest mean disease severity score in both years. Meanwhile, genotypes with LG02_Seyval blanc_/LG15_MN1069_ had intermediate mean score between those with MN1069-inherited haplotypes and those with LG02_MN1069_/LG15_Seyval blanc_. Thus, phenotypic correlation with haplotype combinations for the two chromosomal segments was consistent in both years, where vines with ‘Seyval blanc’-inherited haplotypes showed significantly lowest disease severity. Therefore, the evidence suggests the identification of a powdery mildew resistance in grape, henceforth called *Ren10*.

**Fig. 3:**
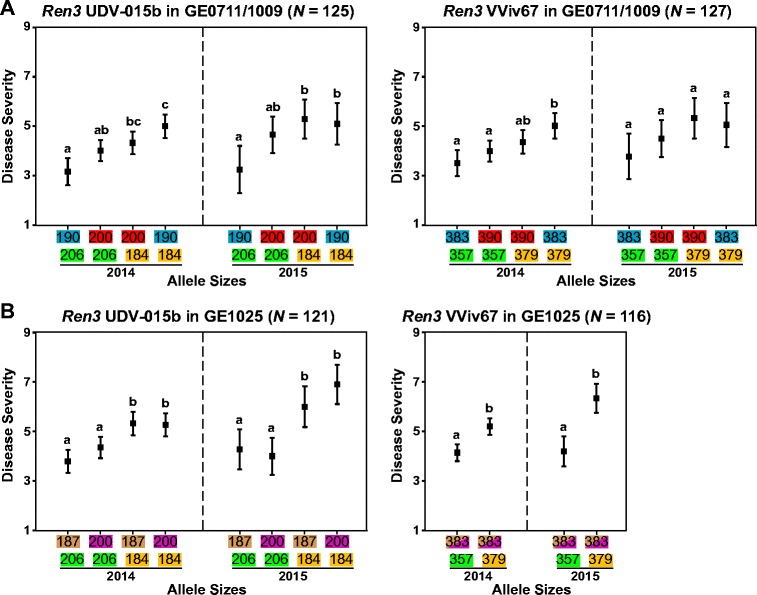
Correlation between disease severity and PCR fragment sizes from the assessment of two SSR markers (UDV-015b and VViv67) that are tightly linked to the *Ren3* locus. Results were shown in 2 years of each bi-parental family. Different *letters* were assigned on score ranges to indicate statistical significance that was computed by Tukey’s HSD test (*p* < 0.05)

Screening each bi-parental mapping family with two SSR markers (UDV-015b and VViv67) with previously demonstrated linkage to *Ren3* (Welter et al. 2007) provided fragment sizes that were associated with powdery mildew resistance (Fig. 3). In the assessment of GE1025 (*N* = 121) with marker UDV-015b, PCR products of MN1264 yielded fragment sizes of 184/206 while MN1246 (male parent) produced fragment sizes of 187/200, thereby resulting in four combinations of allele sizes in the offspring (Fig. 1). ANOVA and Tukey’s HSD test indicated that individuals containing the 206-bp fragment exhibited significantly reduced disease severity in both years (Fig. 3). In GE0711/1009, meanwhile, MN1214 (male parent) produced fragment sizes of 190/200, which also resulted in four combinations of allele sizes in the offspring (*N* = 125). Though a similar trend was observed in both years, ANOVA and Tukey’s HSD test showed statistically significant association of the 206-bp fragment with resistance only when it was paired with the MN1214-derived 190-bp fragment.

Meanwhile, PCR products of VViv67 in GE1025 (*N* = 116) yielded fragment sizes of 357/379 in MN1264 and homozygous 384/384 in MN1246, providing two unique combinations of allele sizes in the offspring (Fig. 1). In both years, ANOVA and Tukey’s HSD test demonstrated an association between the 357-bp fragment and powdery mildew resistance. Meanwhile, PCR products of MN1214 (male parent) reported fragment sizes of 383/390, resulting in four combinations of allele sizes in the offspring of GE0711/1009 (*N* = 127). However, ANOVA calculations revealed statistically significant association of the 357-bp fragment with resistance only in year 2014, but not in 2015.

### Haploblock validation using a controlled inoculation experiments

The phenotypic responses from two independent controlled inoculation experiments were used to compare the four haplotype combinations. Based on the TransectT results in experiment A, offspring with the ‘Seyval blanc’-inherited *Ren10* haplotype exhibited a statistically lower hyphal proliferation than offspring with either the MN1069-inherited *Ren10* or the ‘Seyval blanc’-inherited *Ren3* haplotype (Fig. 2b; Table S3b).

The results from hyphal transects experiments (Table S3a) were comparable to TransectT, although heteroscedasticity analyses reported by Cadle-Davidson et al. (2016) indicated the need for a cube root transformation for a more uniform distribution of variance. In the case of sporulation, individuals with the ‘Seyval blanc’-inherited *Ren10* haplotype had a statistically lower incidence of sporulation than offspring with either the MN1069-inherited *Ren10* or the ‘Seyval blanc’-inherited *Ren3* haplotype (Table S3c). This corroborated the results of hyphal transects and TransectT.

Meanwhile, polymorphism for LG 15 haplotype showed no significant effect on either colonization (*p* value = 0.1459) or sporulation (*p* value = 0.4607). Results in experiment B appeared to have a systematic shift (*p* value <1 × 10^−4^), where values in all haplotype combinations were shifted upward by approximately 0.7–0.9 units. While significantly more disease was observed across the second experiment, the allele effect was consistent over both experiments, as shown for TransectT (Fig. 2b), and all experiment × haplotype interaction terms were non-significant at *α* = 0.1 (Table S3).

## Discussion

### Genotyping: sequencing and SNP assignment

The percentage of GBS tag alignment (∼67 %) and the number of putative SNP markers yielded (521,405 SNPs) for the 436 individuals considered in this study were similar to the reports of other families that were included in the *Vitis*Gen project and analyzed by Hyma et al. (2015). Although our investigation utilized a corrected version of the *V. vinifera* reference genome PN40024 12×.v2 (Adam-Blondon et al. 2011, Jaillon et al. 2007), several reads did not align likely due to divergent species-specific sequences, low-complexity sequences and poor quality, as previously suggested by Barba et al. (2014) and Hyma et al. (2015). The confidence in assigning heterozygote marker genotypes was dependent on the depth of sequence coverage (Hyma et al. 2015), a known issue happening with GBS while analyzing heterozygous individuals that affects our ability to obtain reliable pseudo-testcross markers for linkage map construction. In a bi-parental analysis of families with complex genetic backgrounds (as in our case, which includes at least six *Vitis* species), the optimal combination of GBS multiplexing, read depth, and the number of SNPs has to be assessed beforehand, as suggested for another heterozygous perennial species, *Populus trichocarpa* (Schilling et al. 2014).

### Parental and consensus maps

In a cold-climate region, a grapevine breeding program may employ recurrent selection to select for winter hardiness and maturity time, as well as other important fruit quality traits. Due to the contribution of different trait alleles by various *Vitis* species, this selection method often results in experimental families with ancestry comprised of numerous *Vitis* species. To date, many grapevine genetic maps that have been published were based either on *V. vinifera* intra-specific crosses (Adam-Blondon et al. 2004; Doligez et al. 2002; Doligez et al. 2006; Riaz et al. 2004; Troggio et al. 2007), or hybrids of interspecific crosses involving up to three *Vitis* species (Barba et al. 2014; Bellin et al. 2009; Blasi et al. 2011; Dalbó et al. 2000; Di Gaspero et al. 2007; Doucleff et al. 2004; Fischer et al. 2004; Grando et al. 2003; Lodhi et al. 1995; Lowe and Walker 2006; Marguerit et al. 2009; Moreira et al. 2010; Salmaso et al. 2008; Welter et al. 2007). This paper reports a grapevine genetic map based on hybrids of crosses between parents derived from at least six *Vitis* species.

The high heterozygosity in grapevine necessitates the use of a pseudo-testcross strategy for F_1_ map construction, which builds each parental map independently. Comparisons of the two maternal genetic maps and the consensus map (Fig. S4) revealed general similarity in map length, marker order, and LG sizes, except for minor discrepancies, namely putative inversion segments at a few regions, particularly in LG 15 and LG 16. Plausible explanations for these discrepancies include the use of relatively small family sizes and technical challenges of using GBS for genotypes with multiple species backgrounds. In addition, the region with several markers in inverted order may be attributed to the presence of a large cluster of NBS-LRR genes comprising *Ren3* (Welter et al. 2007).

In the maternal maps, a region on chromosome 15 exhibited significant segregation distortion, an observation previously reported by Lowe and Walker (2006), Welter et al. (2007), Riaz et al. (2011), Heerden et al. (2014) and Guo et al. (2014). In contrast, the paternal maps (Figs. S5 and S6) had more prominent challenges, perhaps due to decreased sampling of parental meioses. In particular, the genetic map of MN1246 included two short unconnected fragments of LG 15, as well as the absence of LG 17. Meanwhile, the map of MN1214 contained several sizeable gaps, particularly in LG 1 and LG 8. These limitations could be due to biological reasons (e.g., lack of polymorphism due to high levels of homozygosity, or the presence of lethal alleles which results in a lethal phenotype in homozygous condition) and/or technical reasons (e.g., sequencing quality, unsuitable reference genome for these families, missing data, or stringent calls).

### Field assessment of powdery mildew severity

In a cold-climate region where breeding populations are potentially subjected to low-temperature injury each year, winter injury to a vine or differences in overwintering success of the pathogen may affect the field evaluation of disease severity in the following growing season as well as weather conditions during the growing season. Here, resistance to powdery mildew displayed a relatively continuous variation in the two experimental F_1_ families in both years, suggesting inheritance of a quantitative trait (Fig. S1). However, in 2014, the distribution appeared distinctly bimodal, suggesting the presence of loci with major effects. Despite the year-to-year changes in the severity of symptoms and rating scales used, we reported consistent and reproducible QTL results.

### QTL detection and validation

Pedigree-connected multi-parental families can be comprised of several bi-parental families that are connected by a common parent. In comparison to single bi-parental family analyses, the use of joint analyses has proved to optimize QTL detection in carrot (Le Clerc et al. 2015), maize (Blanc et al. 2006; Steinhoff et al. 2011), oil palm (Billotte et al. 2010), perennial ryegrass (Pauly et al. 2012), and sugar beet (Schwegler et al. 2013). While a single bi-parental family takes into account only two alleles from each parent and allelic effects are specific to the family, connected multi-parental families can provide estimates for the allelic effects of different parental lines. As evidenced from our investigation, the joint analyses provided validation and higher precision than separate analyses for QTL locations, resulting in finer support intervals shown in both IM and CIM analyses.

In this study, we reported the identification of a major QTL on LG 15 and a moderate QTL on LG 2. A QTL named *Ren3* was previously identified on LG 15 (Welter et al. 2007), but to our knowledge, a QTL for powdery mildew resistance has not previously been reported on LG 2, and thus we name this resistance locus, resistance to *E. necator 10* (*Ren10*). Justification for naming this locus is also supported by haploblock validation, which is described in the ensuing section. The QTLs on LGs 15 and 2 explained up to 29.4 and 13.3 % of the total phenotypic variance, respectively. The relatively modest percentages of variance explained may be due to the experimental design of using a single unreplicated vine of each individual, as well as uneven distribution of inoculum, varying environmental conditions in the field, and other QTL not detected in our experimental design. Despite the detection of a major QTL on LG 15, this QTL was not detected in GE0711/1009 in year 2015. This could be attributed to the vine age and smaller sample size of GE0711.

Our QTL analyses also included the use of AUDPC as a phenotype. The collection of field ratings over multiple time points throughout the growing season was hypothesized to enhance QTL detection, since AUDPC would likely capture more phenotypic variance than that of a single time-point rating. However, the QTL results in both years identified the same QTL as a single late-season rating, without providing additional utility or resolution. Therefore, AUDPC corroborated the single time-point ratings. However, for the conditions and goals of the breeding program addressed here, the use of AUDPC does not seem profitable due to the additional time and efforts incurred, and it did not provide further information about the genetic control of powdery mildew resistance.

Despite GE0711/1009 and GE1025 sharing a common maternal parent, LG 2 QTL was detected in the former, but not in the latter, which was likely attributed to the allelic contribution of the different male parents. The male parents of GE0711/1009 and GE1025 (i.e., MN1214 and MN1246, respectively) are half-sibs, sharing ‘Frontenac’ as a common parent. However, the non-common parents of MN1214 and MN1246 are *V. vinifera* cv. Cabernet Sauvignon and *Vitis* hybrid MN1200, respectively, where the former is highly susceptible to powdery mildew, while the latter is moderately resistant to the disease. Thus, our ability to detect the maternal QTL on LG 2 may be due to the enrichment for paternally derived susceptible alleles in GE0711/1009 from the grandparent ‘Cabernet Sauvignon’.

Coincidentally, chromosomes 2, 15, and 16 belong to the same cluster of paralogs that demonstrated the contribution of three ancestral genomes to the present-day grapevine haploid genome (Jaillon et al. 2007). Thus, the two QTLs on LGs 2 and 15 may have arisen from either a hexaploidization event, or through two successive genome duplications. Subsequent to the polyploidization event, nonrandom loss of genomic content likely led to preferential retention of genic regions that were associated with transcription factors, as well as expressions of novel morphologies and pathogen resistance (Doyle et al. 2008).

### Haplotypes associated with powdery mildew resistance

Development of SNP-based haplotypes that are associated with QTL alleles is useful for quantifying their effects and may provide DNA information for marker-assisted selection of parents or seedlings. Our analysis identified the desirable genetic contribution owing to a specific grandparent, ‘Seyval blanc’. Based on the two QTLs, four unique maternal haplotype combinations were constructed spanning ∼6.6 cM on LG 2 and ∼24.3 cM on LG 15 using SNPs from the maternal maps. In 2 years of observations, the presence of ‘Seyval blanc’-inherited haplotypes was significantly associated with decreased disease severity, while offspring with MN1069-inherited haplotypes exhibited significantly higher disease scores. This analysis reaffirmed the relatively larger effect of the QTL on LG 15 compared to the QTL on LG 2.

Meanwhile, laboratory controlled inoculation of the GE1025 mapping families provided strong support for the discovery of the QTL on LG 2. In two independent experiments measuring three phenotypic responses, individuals with the ‘Seyval blanc’-inherited *Ren10* haplotype consistently exhibited reduced colonization and decreased sporulation. The use of a single isolate (*E. necator* NY19) that originated from New York *V. vinifera* vineyards (Cadle-Davidson, personal communication) allowed the detection of the LG 2 QTL, which was more difficult to detect in the field due to the larger effect of the LG 15 QTL, and/or the genetics of the pathogen population in the natural field environment. *Ren3* resistance is known to be ineffective in New York (Cadle-Davidson and Reisch, personal communication), and thus it is not surprising that *Ren3* was not detected using the NY19 isolate.

### Co-localization with *Ren3*?

The detection of a major QTL on LG 15 raised a question of whether this QTL co-localizes with the previously reported *Ren3* locus (Welter et al. 2007). Although we are provisionally designating it as *Ren3*, additional experiments such as complementation tests using both sources of resistance in this region will have to be conducted to address this question.

We screened our mapping families with two SSR markers, namely UDV-015b and VViv67 that were reportedly linked with *Ren3* (Welter et al. 2007) to establish a correlation between specific fragment sizes and disease severity in our families. The 206-bp fragment from UDV-015b and the 357-bp fragment from VViv67 were significantly associated with reduced mean powdery mildew severity. Though these correlations were consistently significant in GE1025 in both years, the resistant alleles in GE0711/1009 trended toward the expected effects in all cases, except the lack of association between fragment sizes and disease severity in 2015. Given the vine age of GE0711 mentioned above, and the use of field phenotypes, variance due to environment was likely larger than variance attributed to genetics. As a whole, our results have shown that the UDV-015b and VViv67 markers that were developed for the *Ren3* locus appear linked to resistance inherited from MN1264, and were ultimately derived from ‘Seyval blanc’.

### Chromosome 2: *Ren10* and potential structural dynamics in the vicinity

The identification of the *Ren10* powdery mildew resistance locus on chromosome 2 is intriguing, given the proximal location of several *Myb*-like genes controlling berry color, at a physical position of approximately 14.2 Mbp in the *V. vinifera* reference genome (Jaillon et al. 2007; Velasco et al. 2007; Walker et al. 2006). This region has been characterized by movement of the *Gret1* retrotransposon and by hemizygosity in some genotypes due to chromosomal replacement and/or deletion (Kobayashi et al. 2004, 2005; Lijavetzky et al. 2006; Migliaro et al. 2014; Pelsy et al. 2015; Walker et al. 2007).

Here, the putative physical position of *Ren10*, which was likely introgressed from a North American species, resides in the QTL window between 8.5 and 18.7 Mbp (Table S2). In the GE1025 mapping family, a berry color locus was mapped to a physical position from 10.6 to 17.6 Mbp (Clark et al. 2016). To test whether the *Ren10* QTL spans structural variations related to a berry color locus, we re-sequenced MN1264 and MN1246, the parents of GE1025. The results indicate several regions of hemizygosity in this region (Fig. S7). In particular, the physical position 10.5–12.5 Mbp is single copy in MN1246, suggesting hemizygosity, and is completely absent in MN1264, likely due to a homozygous deletion. This preliminary analysis is solely based on alignment to the *V. vinifera* reference genome, and thus additional genomic characterizations need to be conducted.

## Conclusion

At the University of Minnesota grape breeding program, MN1264 is a quality hybrid wine grape that is unfortunately not suited for production due to the production of only female flowers. However, MN1264 has various favorable traits, such as winter hardiness, fungal resistance, and pest resistance, thus making it an ideal crossing parent for genetic studies within the context of a breeding program (Luby, personal communication). In an effort to study the genetic determinism of powdery mildew resistance, MN1264 was used to construct two segregating mapping families, which are described in this study.

Here, we present the first application of joint-family SNP-based analysis in grapevine to construct a consensus map for QTL identification and validation, the development of haplotypes, as well as the report of resistance-linked fragment sizes from SSR markers. First, the use of GBS provided over 10,000 uniformly distributed genome-wide SNPs, of which 2732 were used to generate high-density genetic maps. Next, a robust consensus map was developed, and then QTL analyses were performed to corroborate marker-trait association test results using 2 years of field phenotypic data. We detected a major QTL on LG 15 that may co-localize with the previously reported *Ren3* locus (Di Gaspero et al. 2005; Merdinoglu et al. 2005; Welter et al. 2007), which allowed the use of closely linked SSR markers to screen the mapping families. In addition, a novel QTL (*Ren10*) with moderate effect was detected and this discovery was validated with a controlled inoculation study. Finally, the availability of high-quality SNP markers allowed development of haplotypes associated with the QTLs to identify grandparental origin of powdery mildew resistance. In the case of *Ren3*, the use of linked SSR markers established a correlation between fragment sizes with resistance-carrying haplotypes that were not previously reported. This enables marker-assisted breeding for powdery mildew resistant individuals. Meanwhile, for the *Ren10* QTL, fine mapping and additional marker development has to be made to convert the locus into reliable DNA markers before marker-assisted breeding can be applied effectively. The use of these molecular markers has the potential of helping breeders develop cultivars with more durable resistance to the worldwide economically damaging powdery mildew.

Within the larger context of our study, we demonstrated the advantage of dissecting complex genetic backgrounds that enabled the discovery of a novel resistance locus. Given the vast diversity in grape that has mostly been underexplored, future studies should explore other grape species to uncover potentially novel sources of resistance in an effort to develop resistant cultivars.

## Electronic supplementary material

Fig. S1 Two-dimensional histogram and scattered distribution of field powdery mildew severity in 2 years. Raw phenotypic information was jittered to show overlapping data points (JPEG 278 kb)

High-resolution image (EPS 1291 kb)

Fig. S2 Distribution of field severity of powdery mildew in 2014 and 2015. (JPEG 546 kb)

High-resolution image (EPS 1758 kb)

Fig. S3 QTL results for powdery mildew resistance on LG 2 and LG 15 of the maternal maps of GE0711/1009 and GE1025, as well as the consensus map in both years using IM and CIM analyses. Genetic regions, where *colorized*, indicated intervals that exceeded a statistical threshold value of 3.0. LOD scores were color-coded as described in the legend. (JPEG 1526 kb)

High-resolution image (EPS 4388 kb)

Fig. S4 Genetic maps of MN1264. At each LG, the consensus map (*middle*) was constructed using maternal information from GE0711/1009 (*left*) and from GE1025 (*right*). (JPEG 15241 kb)

High-resolution image (EPS 7269 kb)

Fig. S5 A genetic map of MN1214 or paternal map of GE0711/1009. (JPEG 2012 kb)

High-resolution image (EPS 6000 kb)

Fig. S6 A genetic map of MN1246, or paternal map of GE1025. The map lacks LG 17, and contains two LG 15 segments that could not be joined. (JPEG 1463 kb)

High-resolution image (EPS 5995 kb)

Fig. S7 Distribution of the fraction of sequence reads with respect to the per base coverage read depth for the individuals: **a** MN1264; **b** MN1246 in the *Ren10* QTL region. Each *solid line* represents the per base coverage read depth distribution for a 2-Mbp section within the *Ren10* region. The *black line* represents the genome-wide per base coverage read depth distribution. The data come from a parallel project for the whole genome re-sequencing of parental germplasm within *Vitis*Gen. (JPEG 525 kb)

High-resolution image (EPS 976 kb)

ESM 1 (DOCX 16 kb)

ESM 2 (DOCX 27 kb)

ESM 3 (DOCX 20 kb)
