# Supplementary figures and images for: Genetic dissection of powdery mildew resistance in interspecific half-sib grapevine families using SNP-based maps
Source: Mol Breed. 2016 Dec 21;37(1):1. doi: 10.1007/s11032-016-0586-4 (PMC5226326; doi:10.1007/s11032-016-0586-4)

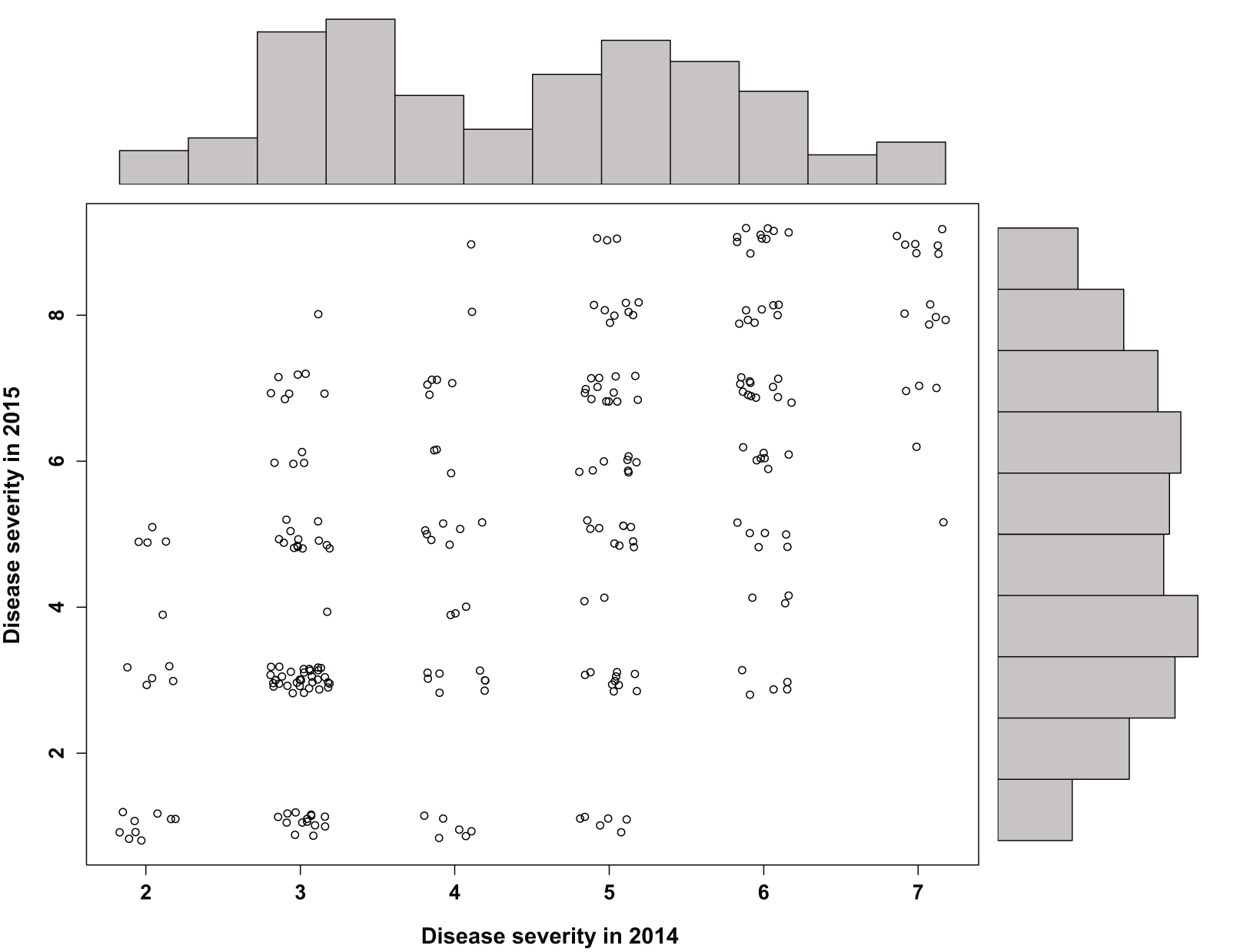

Supplement: Supplementary file 1 — Two-dimensional histogram and scattered distribution of field powdery mildew severity in 2 years. Raw phenotypic information was jittered to show overlapping data points (JPEG 278 kb) [file 11032_2016_586_Fig4_ESM.jpg]

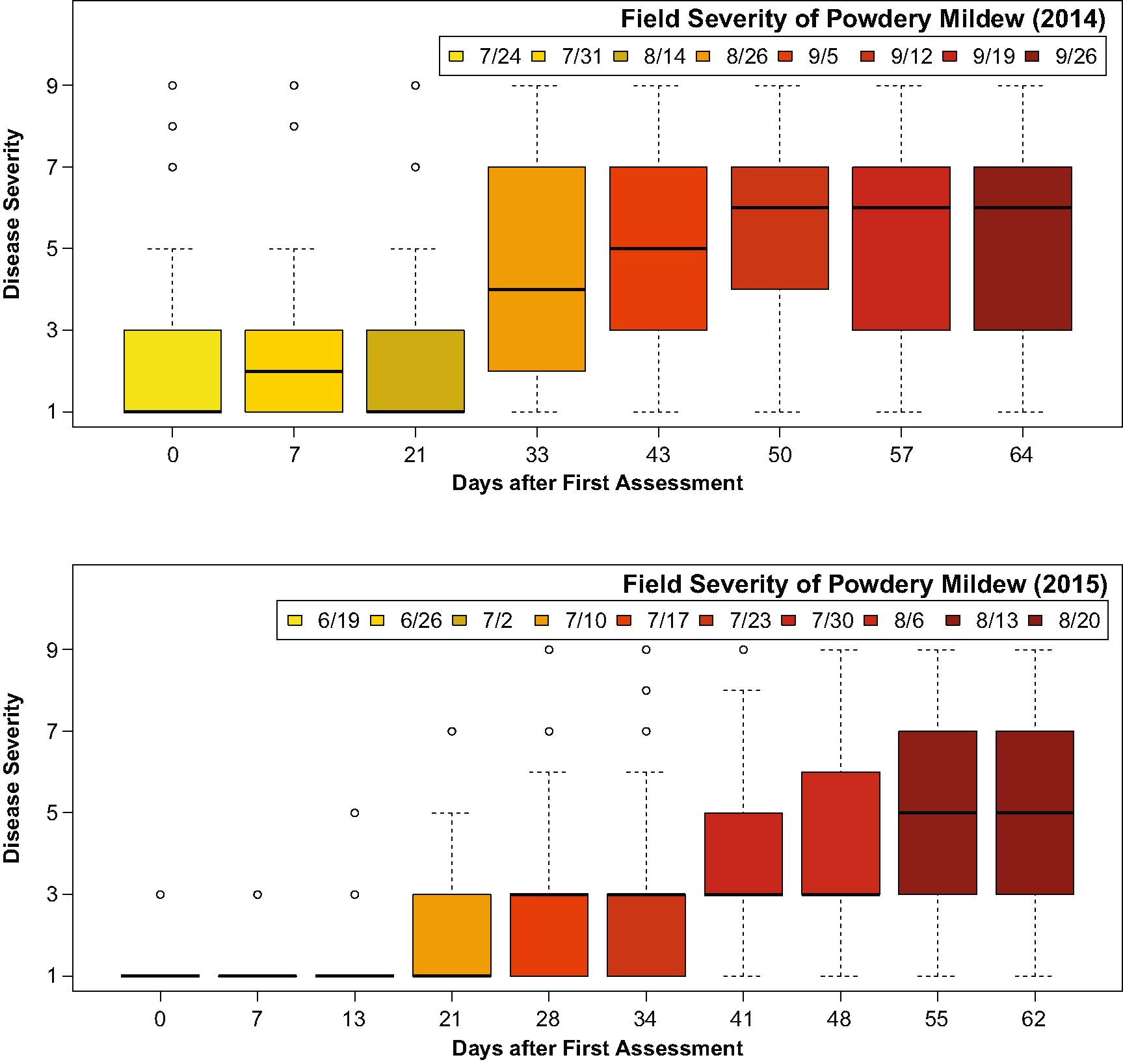

Supplement: Supplementary file 3 — Distribution of field severity of powdery mildew in 2014 and 2015. (JPEG 546 kb) [file 11032_2016_586_Fig5_ESM.jpg]

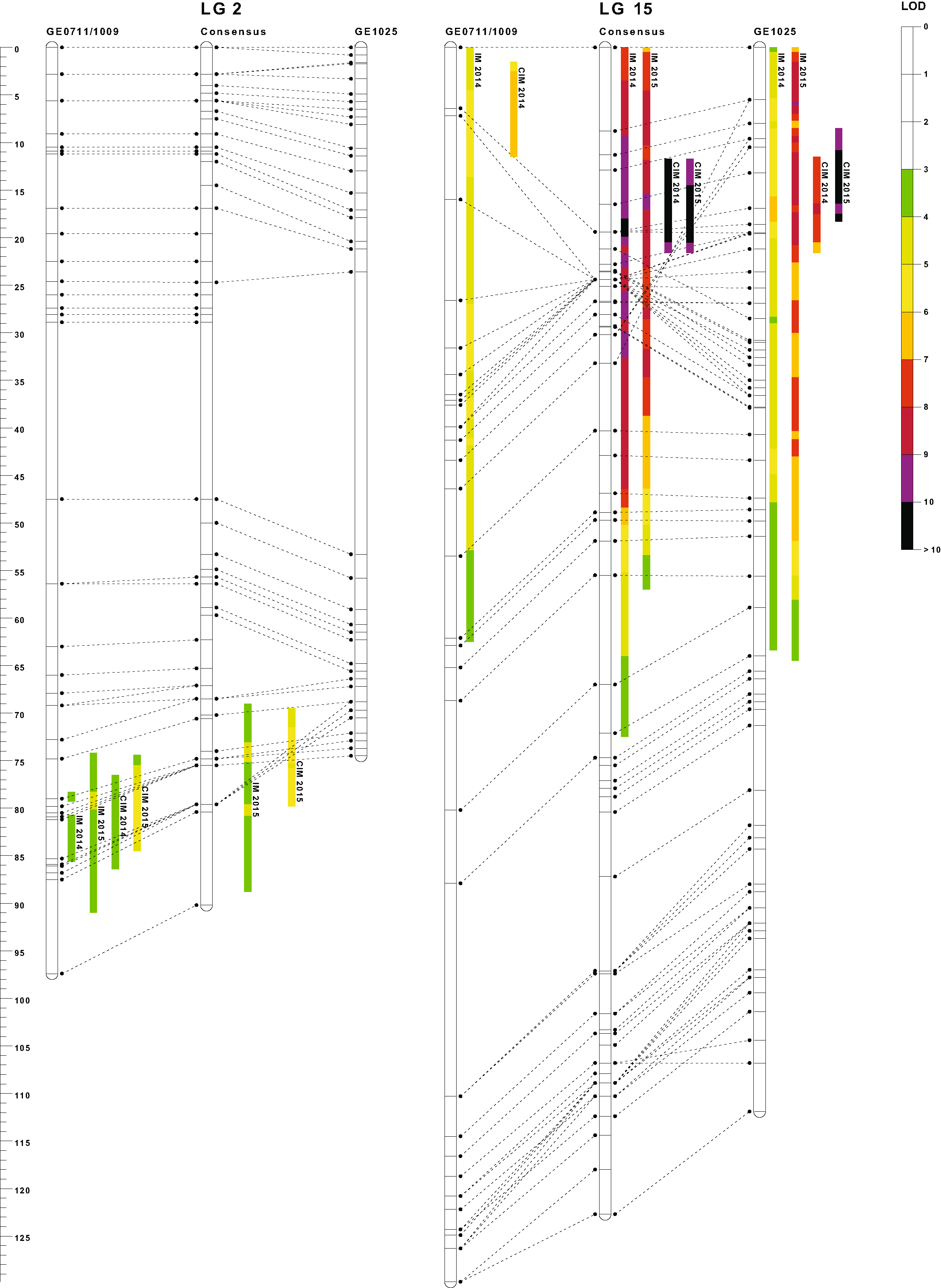

Supplement: Supplementary file 5 — QTL results for powdery mildew resistance on LG 2 and LG 15 of the maternal maps of GE0711/1009 and GE1025, as well as the consensus map in both years using IM and CIM analyses. Genetic regions, where colorized, indicated intervals that exceeded a statistical threshold value of 3.0. LOD scores were color-coded as described in the legend. (JPEG 1526 kb) [file 11032_2016_586_Fig6_ESM.jpg]

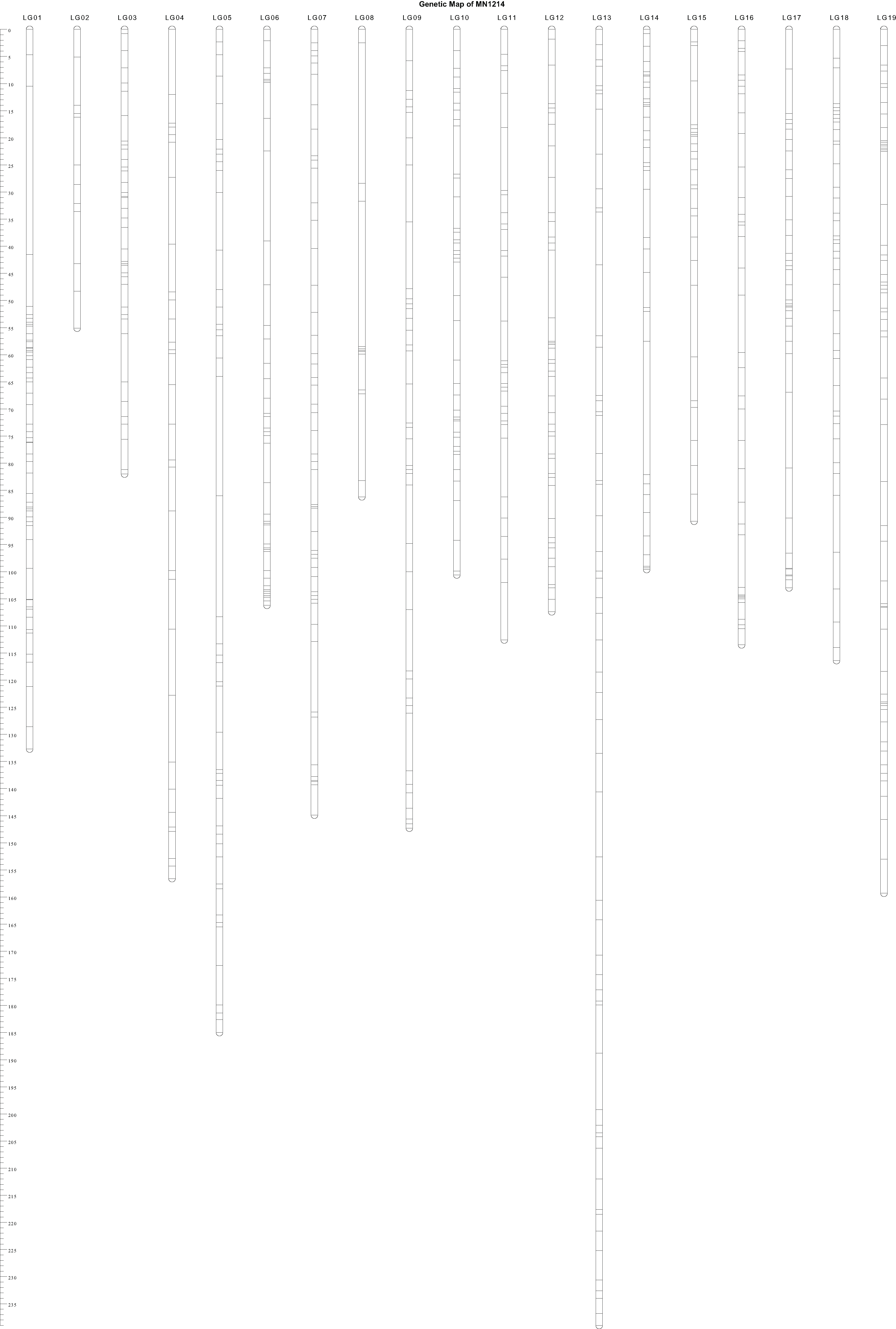

Supplement: Supplementary file 9 — A genetic map of MN1214 or paternal map of GE0711/1009. (JPEG 2012 kb) [file 11032_2016_586_Fig8_ESM.jpg]

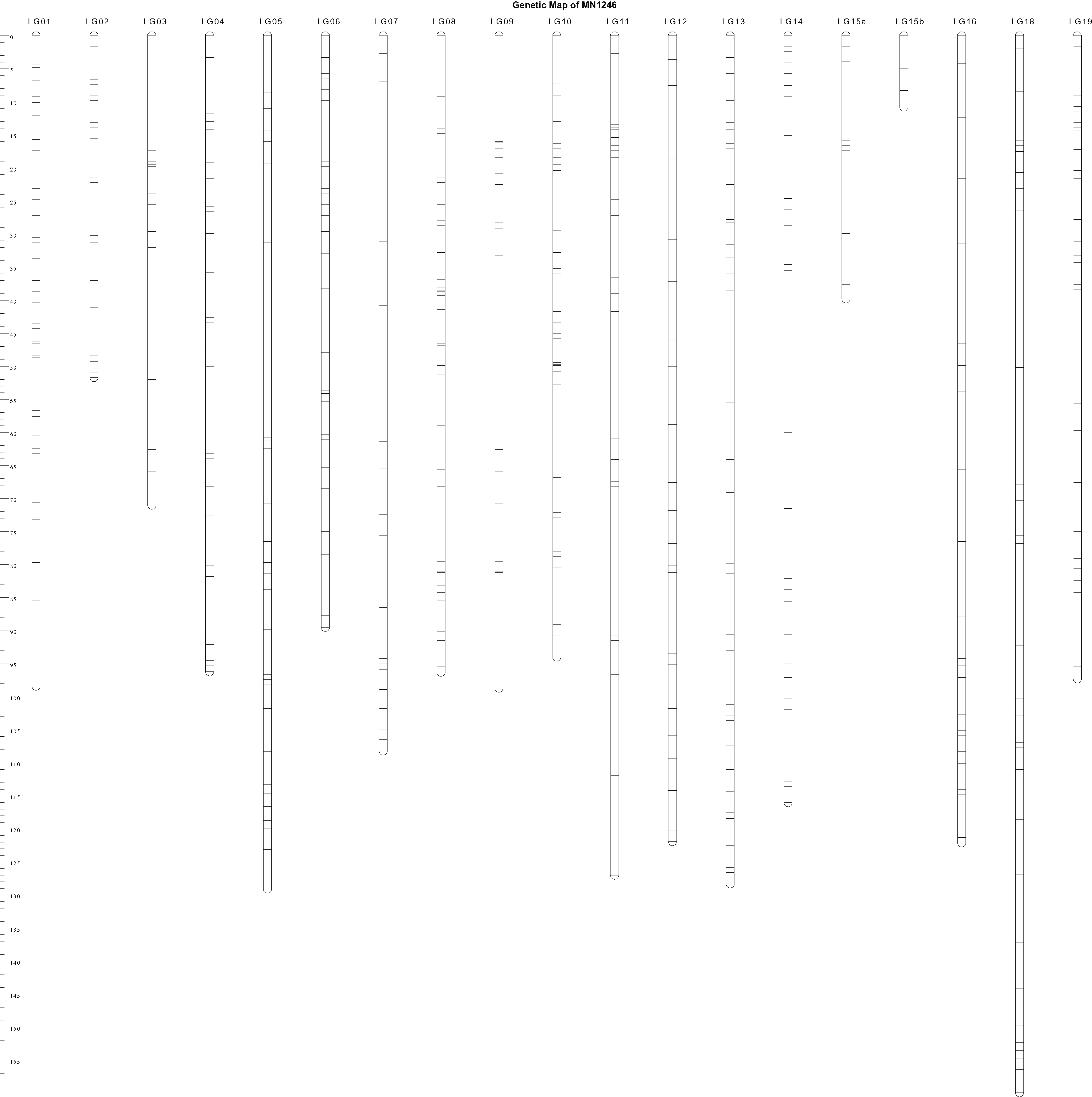

Supplement: Supplementary file 11 — A genetic map of MN1246, or paternal map of GE1025. The map lacks LG 17, and contains two LG 15 segments that could not be joined. (JPEG 1463 kb) [file 11032_2016_586_Fig9_ESM.jpg]

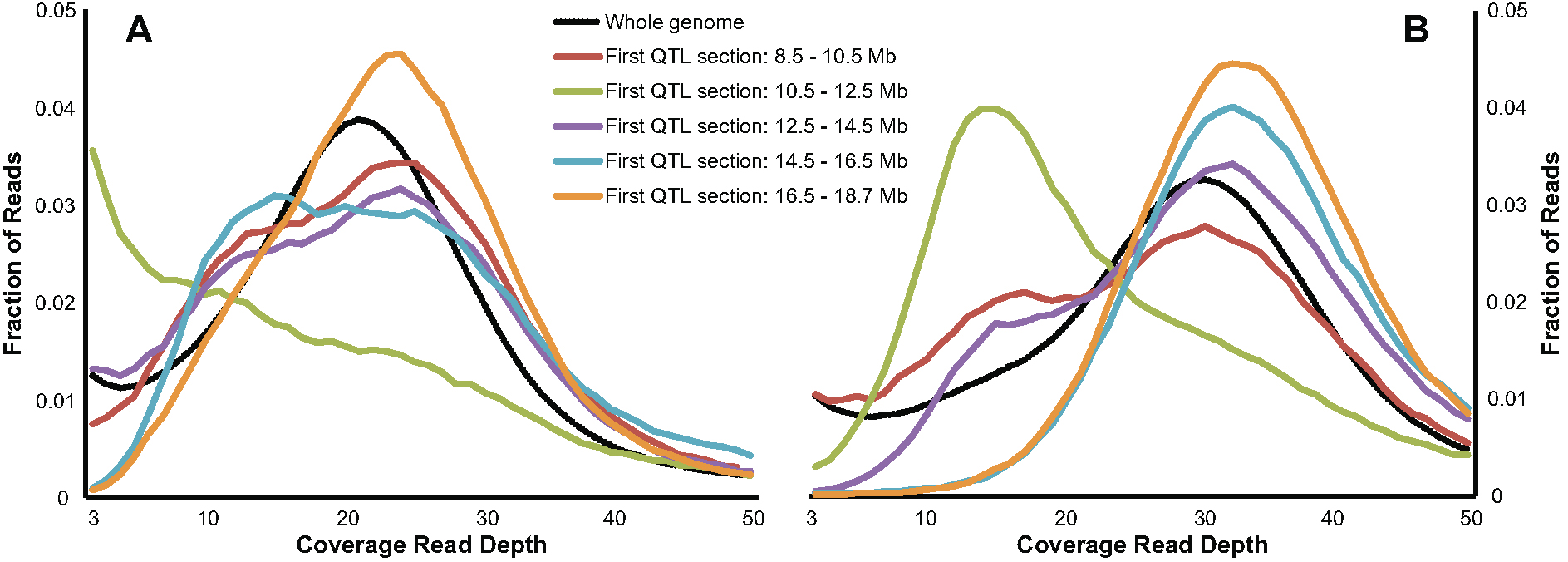

Supplement: Supplementary file 13 — Distribution of the fraction of sequence reads with respect to the per base coverage read depth for the individuals: a MN1264; b MN1246 in the Ren10 QTL region. Each solid line represents the per base coverage read depth distribution for a 2-Mbp section within the Ren10 region. The black line represents the genome-wide per base coverage read depth distribution. The data come from a parallel project for the whole genome re-sequencing of parental germplasm within VitisGen. (JPEG 525 kb) [file 11032_2016_586_Fig10_ESM.jpg]
