# Supplementary material for: Genetic dissection of powdery mildew resistance in interspecific half-sib grapevine families using SNP-based maps
Source: Mol Breed. 2016 Dec 21;37(1):1. doi: 10.1007/s11032-016-0586-4 (PMC5226326; doi:10.1007/s11032-016-0586-4)
Supplement: Supplementary file 15 — (DOCX 16 kb) [file 11032_2016_586_MOESM8_ESM.docx]

**Genetic dissection of powdery mildew resistance in interspecific half-sib grapevine families using SNP-based maps**

***Molecular Breeding***

*Electronic Supplementary Material 1*

Soon Li Teh^1^, Jonathan Fresnedo-Ramírez^2^, Matthew D. Clark^1^, Qi Sun^2^, Lance Cadle-Davidson^3^, James J. Luby^1^

^1^ Department of Horticultural Science, University of Minnesota, Saint Paul, MN 55108

^2^ BRC Bioinformatics Facility, Institute of Biotechnology, Cornell University, Ithaca, NY 14853

^3^ USDA-ARS Grape Genetics Research Unit, Geneva, NY 14456

Corresponding author:

James J. Luby

Department of Horticultural Science, University of Minnesota, Saint Paul, MN 55108

Email: lubyx001@umn.edu

**Supplementary Table S1** Number of SNPs and total genetic distance (cM) of linkage groups (LGs) in paternal genetic maps of GE0711/1009 (MN1214) and GE1025 (MN1246). The MN1246 map lacks LG 17, and contains two LG 15 segments that could not be merged.

| LG | MN1214 | | MN1246 | |
| --- | --- | --- | --- | --- |
|  | Number of SNPs | Total genetic distance (cM) | Number of SNPs | Total genetic distance (cM) |
| 1 | 101 | 132.7 | 120 | 98.4 |
| 2 | 16 | 55.1 | 49 | 51.7 |
| 3 | 66 | 82.0 | 47 | 71.0 |
| 4 | 54 | 156.6 | 61 | 96.2 |
| 5 | 83 | 185.0 | 99 | 129.1 |
| 6 | 93 | 106.2 | 92 | 89.5 |
| 7 | 75 | 144.9 | 41 | 108.2 |
| 8 | 24 | 86.2 | 81 | 96.3 |
| 9 | 68 | 147.3 | 58 | 98.7 |
| 10 | 70 | 100.6 | 97 | 94.0 |
| 11 | 55 | 112.6 | 67 | 127.0 |
| 12 | 69 | 107.4 | 63 | 121.9 |
| 13 | 92 | 239.0 | 112 | 128.3 |
| 14 | 70 | 99.6 | 67 | 116.0 |
| 15 | 45 | 90.7 | 45^a^ | 50.5^a^ |
| 16 | 54 | 113.5 | 72 | 122.1 |
| 17 | 76 | 103.0 | – ^b^ | – ^b^ |
| 18 | 63 | 116.4 | 108 | 159.9 |
| 19 | 90 | 159.3 | 96 | 97.3 |
| Total | 1264 | 2338.1 | 1375 | 1856.1 |

^a^ Numbers were expressed as a sum of two LG 15 segments.

^b^ The paternal map of GE1025 was unable to identify a linkage group that corresponded to chromosome 17.
