# Supplementary material for: Genetic dissection of powdery mildew resistance in interspecific half-sib grapevine families using SNP-based maps
Source: Mol Breed. 2016 Dec 21;37(1):1. doi: 10.1007/s11032-016-0586-4 (PMC5226326; doi:10.1007/s11032-016-0586-4)
Supplement: Supplementary file 16 — (DOCX 27 kb) [file 11032_2016_586_MOESM9_ESM.docx]

**Genetic dissection of powdery mildew resistance in interspecific half-sib grapevine families using SNP-based maps**

***Molecular Breeding***

*Electronic Supplementary Material 2*

Soon Li Teh^1^, Jonathan Fresnedo-Ramírez^2^, Matthew D. Clark^1^, Qi Sun^2^, Lance Cadle-Davidson^3^, James J. Luby^1^

^1^ Department of Horticultural Science, University of Minnesota, Saint Paul, MN 55108

^2^ BRC Bioinformatics Facility, Institute of Biotechnology, Cornell University, Ithaca, NY 14853

^3^ USDA-ARS Grape Genetics Research Unit, Geneva, NY 14456

Corresponding author:

James J. Luby

Department of Horticultural Science, University of Minnesota, Saint Paul, MN 55108

Email: lubyx001@umn.edu

**Supplementary Table S2** Genotypes, QTL-spanning SNPs for LG 2 and LG 15, as well as field powdery mildew phenotypes for haplotype construction. SNPs from maternal genetic maps were used to trace the inheritance of resistant haplotypes from the maternal grandparents (i.e. 'Seyval blanc' and MN1069). At each locus, ‘A’ or ‘B’ was denoted to indicate inheritance from 'Seyval blanc' or MN1069, respectively. The order of the SNPs is presented with respect to the maternal genetic maps.

| **Genotype** | **SNPs on LG 2** | | | | | | | | | | | | **SNPs on LG 15** | | | | | | | | **Field PM Phenotype** | |
| --- | --- | --- | --- | --- | --- | --- | --- | --- | --- | --- | --- | --- | --- | --- | --- | --- | --- | --- | --- | --- | --- | --- |
|  | **S2_8538108** | **S2_14108168** | **S2_14108170** | **S2_16681176** | **S2_17017148** | **S2_15954410** | **S2_16681183** | **S2_15052983** | **S2_17854965** | **S2_18455557** | **S2_18507119** | **S2_18702143** | **S15_2190957** | **S15_1931143** | **S15_102583** | **S15_202858** | **S15_98213** | **S15_98184** | **S15_584141** | **S15_1308526** | **2014**^a^ | **2015**^b^ |
| GE0711_005 | B | B | B | B | B | B | B | B | B | B | B | B | B | –^c^ | B | B | B | B | B | B | 7 | 8 |
| GE0711_026 | B | B | B | B | B | B | B | B | B | B | B | B | B | – | B | B | B | B | B | B | 6 | 8 |
| GE1009_048 | B | B | B | B | B | B | B | B | B | B | B | B | B | B | B | B | B | B | B | B | 7 | 9 |
| GE1009_049 | B | B | B | B | B | B | B | B | B | B | B | B | B | B | B | B | B | B | B | B | 7 | 8 |
| GE1025_044 | B | B | B | B | B | B | B | B | B | B | B | B | B | B | B | B | B | B | B | B | 7 | 9 |
| GE1025_090 | B | B | B | B | B | B | B | B | B | B | B | B | B | B | B | B | B | B | B | B | 7 | 9 |
| GE0711_004 | A | A | A | A | A | A | A | A | A | A | A | A | B | – | B | B | B | B | B | B | 5 | 5 |
| GE0711_011 | A | A | A | A | A | A | A | A | A | A | A | A | B | – | B | B | B | B | B | B | 6 | 9 |
| GE1009_052 | A | A | A | A | A | A | A | A | A | A | A | A | B | B | B | B | B | B | B | B | 4 | 3 |
| GE1009_093 | A | A | A | A | A | A | A | A | A | A | A | A | B | B | B | B | B | B | B | B | 5 | 7 |
| GE1025_018 | A | A | A | A | A | A | A | A | A | A | A | A | B | B | B | B | B | B | B | B | 5 | 8 |
| GE1025_063 | A | A | A | A | A | A | A | A | A | A | A | A | B | B | B | B | B | B | B | B | 5 | 3 |
| GE0711_020 | B | B | B | B | B | B | B | B | B | B | B | B | A | A | A | A | A | A | A | A | 2 | 5 |
| GE0711_055 | B | B | B | B | B | B | B | B | B | B | B | B | A | A | A | A | A | A | A | A | 3 | 1 |
| GE1009_020 | B | B | B | B | B | B | B | B | B | B | B | B | A | A | A | A | A | A | A | A | 3 | 5 |
| GE1009_050 | B | B | B | B | B | B | B | B | B | B | B | B | A | A | A | A | A | A | A | A | 3 | 5 |
| GE1025_081 | B | B | B | B | B | B | B | – | B | B | B | B | A | A | A | A | A | A | A | A | 3 | 3 |
| GE1025_139 | B | B | B | B | B | B | B | B | B | B | B | B | A | A | A | A | A | A | A | A | 3 | 3 |
| GE0711_033 | A | A | A | A | A | A | A | A | A | A | A | A | A | A | A | A | A | A | A | A | 2 | 1 |
| GE0711_054 | A | A | A | A | A | A | A | A | A | A | A | A | A | A | A | A | A | A | A | A | 2 | 1 |
| GE1009_030 | A | A | A | A | A | A | A | A | A | A | A | A | A | A | A | A | A | A | A | A | 2 | 1 |
| GE1009_127 | A | A | A | A | A | A | A | A | A | A | A | A | A | A | A | A | A | A | A | A | 2 | 1 |
| GE1025_053 | A | A | A | A | A | A | A | A | A | A | A | A | A | A | A | A | A | A | A | A | 3 | 1 |
| GE1025_099 | A | A | A | A | A | A | A | A | A | A | A | A | A | A | A | A | A | A | A | A | 2 | 3 |

^a^ In 2014, vines were assessed using a 7-point visual scale of whole-plant foliage.

^b^ In 2015, vines were assessed using a 9-point IPGRI scale.

^c^ Missing data.
