# Supplementary material for: Genetic dissection of powdery mildew resistance in interspecific half-sib grapevine families using SNP-based maps
Source: Mol Breed. 2016 Dec 21;37(1):1. doi: 10.1007/s11032-016-0586-4 (PMC5226326; doi:10.1007/s11032-016-0586-4)
Supplement: Supplementary file 17 — (DOCX 20 kb) [file 11032_2016_586_MOESM10_ESM.docx]

**Genetic dissection of powdery mildew resistance in interspecific half-sib grapevine families using SNP-based maps**

***Molecular Breeding***

*Electronic Supplementary Material 3*

Soon Li Teh^1^, Jonathan Fresnedo-Ramírez^2^, Matthew D. Clark^1^, Qi Sun^2^, Lance Cadle-Davidson^3^, James J. Luby^1^

^1^ Department of Horticultural Science, University of Minnesota, Saint Paul, MN 55108

^2^ BRC Bioinformatics Facility, Institute of Biotechnology, Cornell University, Ithaca, NY 14853

^3^ USDA-ARS Grape Genetics Research Unit, Geneva, NY 14456

Corresponding author:

James J. Luby

Department of Horticultural Science, University of Minnesota, Saint Paul, MN 55108

Email: lubyx001@umn.edu

**Supplementary Table 3** Statistical analysis of *REN3* and *REN10* prediction of powdery mildew response variables at 9 days post-inoculation following controlled inoculation with *Erysiphe necator* isolate NY19 in two independent experiments.

**Supplementary Table S3a** Report of estimates and confidence intervals of the linear regression for the response variable Hyphal Transects (as a measure of hyphal proliferation), yielded from JMP 12.0.1 (Standard Least Squares personality). This indicates the statistical significance of the predictors Experiment and *REN10*, and the lack of significance (at α ≤ 0.1) of *REN3* and interaction terms in the two experiments with controlled inoculation. In the Term column, in brackets are shown the particular Experiment or origin of the resistance allele for which the estimates were calculated.

| Term | Estimate | S.E.^a^ | t Ratio | Prob > \|t\| | Lower 95% | Upper 95% |
| --- | --- | --- | --- | --- | --- | --- |
| Intercept | 56.63 | 4.08 | 13.88 | <.0001* | 48.63 | 64.64 |
| Experiment[A] | -28.37 | 4.08 | -6.95 | <.0001* | -36.37 | -20.37 |
| *REN10*[MN1069] | 10.84 | 4.10 | 2.64 | 0.0083* | 2.79 | 18.88 |
| *REN3*[MN1069] | -2.19 | 4.09 | -0.54 | 0.5915 | -10.21 | 5.82 |
| Experiment[A]**REN10*[MN1069] | -6.43 | 4.10 | -1.57 | 0.1172 | -14.47 | 1.62 |
| Experiment[A]**REN3*[MN1069] | -2.71 | 4.09 | -0.66 | 0.5081 | -10.72 | 5.31 |

^a^ Standard error.

**Supplementary Table S3b** Report of estimates and confidence intervals of the linear regression for the response variable cube-root transformation of Hyphal Transects (as a measure of hyphal proliferation), yielded from JMP 12.0.1 (Standard Least Squares personality). This indicates the statistical significance of the predictors Experiment and *REN10*, and the lack of significance (at α ≤ 0.1) of *REN3* and interaction terms in the two experiments with controlled inoculation. In the Term column, in brackets are shown the particular Experiment or origin of the resistance allele for which the estimates were calculated.

| Term | Estimate | S.E.^a^ | t Ratio | Prob > \|t\| | Lower 95% | Upper 95% |
| --- | --- | --- | --- | --- | --- | --- |
| Intercept | 1.35 | 0.05 | 26.43 | <.0001* | 1.25 | 1.45 |
| Experiment[A] | -0.40 | 0.05 | -7.87 | <.0001* | -0.50 | -0.30 |
| *REN10*[MN1069] | 0.21 | 0.05 | 4.08 | <.0001* | 0.11 | 0.31 |
| *REN3*[MN1069] | -0.07 | 0.05 | -1.46 | 0.1459 | -0.17 | 0.03 |
| Experiment[A]**REN10*[MN1069] | 0.01 | 0.05 | 0.08 | 0.9347 | -0.10 | 0.10 |
| Experiment[A]**REN3*[MN1069] | -0.04 | 0.05 | -0.75 | 0.4557 | -0.14 | 0.06 |

**Supplementary Table S3c** Report of estimates and confidence intervals of the logistic regression for the response variable sporulation, yielded from JMP 12.0.1 (Standard Least Squares personality). This indicates the statistical significance of the predictors Experiment and *REN10*, and the lack of significance (at α ≤ 0.1) of *REN3* and interaction terms in the two experiments with controlled inoculation. In the Term column, in brackets are shown the particular Experiment or origin of the resistance allele for which the estimates were calculated.

| Term | Estimate | S.E.^a^ | χ^2^ | Prob > χ^2^ | Lower 95% | Upper 95% |
| --- | --- | --- | --- | --- | --- | --- |
| Intercept | 1.19 | 0.06 | 358.88 | <.0001* | 1.06 | 1.31 |
| Experiment[A] | 0.43 | 0.06 | 47.12 | <.0001* | 0.31 | 0.55 |
| *REN10*[MN1069] | -0.24 | 0.06 | 14.52 | 0.0001* | -0.36 | -0.12 |
| *REN3*[MN1069] | 0.05 | 0.06 | 0.54 | 0.4607 | -0.07 | 0.17 |
| Experiment[A]**REN10*[MN1069] | -0.04 | 0.06 | 0.48 | 0.4884 | -0.17 | 0.08 |
| Experiment[A]**REN3*[MN1069] | 0.05 | 0.06 | 0.66 | 0.4160 | -0.07 | 0.17 |
